# Echocardiography as a Predicting Method in Diagnosis, Evaluation and Assessment of Children with Subvalvar Aortic Stenosis

**DOI:** 10.3889/oamjms.2016.016

**Published:** 2016-01-20

**Authors:** Ramush Bejiqi, Hana Bejiqi, Ragip Retkoceri

**Affiliations:** 1 *Division of Cardiology, Pediatric Clinic, University Clinical Center of Kosovo, Prishtina, Republic of Kosovo*; 2 *Main Center of Family Medicine, Prishtina, Republic of Kosovo*

**Keywords:** congenital heart disease, aortic stenosis, subaortic membrane, hypertrophic cardiomyopathy, heart surgery

## Abstract

**BACKGROUND:**

Obstruction to the left ventricular outflow of the heart may be above the aortic valve (5%), at the valve (74%), or in the subvalvar region (23%). These anomalies represent 3 to 6% of all patients with congenital heart defects (CHD), and it occurs more often in males (male-female ratio of 4:1).

**AIM:**

The purpose of this study was to determine the sensitivity and specificity of transthoracic echocardiography in diagnosis of discrete subaortic membrane, to determine convenient time for surgical intervention, and for identifying involvement of the aortic valve by subaortic shelf.

**MATERIAL AND METHODS:**

A retrospective review of the medical records and echocardiograms of 18 patients [14 male (77%) and 4 female (23%)] with discrete subaortic membrane, aged 11 month to 12 years, with mean age of 5 years and 3 month, diagnosed at the Pediatric Clinic in Prishtina, during the period September, 1999 and December, 2010 were done.

**RESULTS:**

Four patients, in neonatal age were operated from critical coarctation of the aorta and, initial signs of congestive heart failure were presented. 2 of them were operated in Belgrade, Serbia and 2 in Lausanne, Switzerland.

**CONCLUSION:**

In all presented patients bicuspid aortic valve was noted, but none of them subaortic membrane was registered.

## Introduction

Obstruction to the left ventricular outflow of the heart may be above the aortic valve (5%), at the valve (74%), or in the subvalvar region (23%). These anomalies represent 3 to 6% of all patients with congenital heart defects (CHD), and it occurs more often in males (male-female ratio of 4:1). Valvar and subvalvar stenosis may be associated with other CHD such as pulmonary stenosis (PS), anomalies of the mitral valve, patent ductus arteriosus (DAP) and, defects of the interatrial septum. A variety of lesion can obstruct the subaortic outflow tract, with or without a coexisting ventricular septal defect. Obstruction can be produced by hypertrophy of the ventricular septum, as seen in hypertrophic cardiomyopathy, by anomalous tissue tags derived from the membranous septum or the leaflets of the atrioventricular valves or by anomalous attachment of the tension apparatus of the atrioventricular valve [1, 2]. When the ventricular septum is intact, the most common lesion is the so-called subvalvar ridge which presents the fibrous shelf that encircles the outflow tract in diaphragmatic fashion. The shelf tends to be a discrete structure, although its position can vary with regard to its proximity to the valvar leaflets. Discrete subaortic stenosis represents a form of subvalvar aortic stenosis characterized by a membranous ridge proximal to the aortic valve. Diagnosis of discrete subaortic stenosis in neonatal period is rare and, lesion more commonly presents as an acquired defect in early infancy or childhood. Two thirds of these patients have associated cardiac lesions, such as ventricular septal defect, DAP or coarctation of the aorta (CoA) [3-6]. Risk of late diagnosis, consequence as a post-operative aortic insufficiency, the potential for recurrent stenosis, and surgical management of discrete subaortic stenosis have traditionally fairly conservative due to the unpredictable rate of progression, postponing resection unit a certain left ventricular outflow tract gradient has been reached.

The most commonly described sequela in patients with subaortic stenosis is aortic regurgitation, which have been estimated to occur to some degree in 30 % to 50 % of pediatric patients and as many as 80 % of adult patients. Damage of aortic valve and subsequent regurgitation is thought to be secondary to the subvalvar high-velocity systolic jet produced by the outflow tract obstruction [3, 4]. Earlier diagnosis and surgical treatment of discrete subaortic stenosis is now preferred by many surgical centers in an effort to avoid consequences such are left ventricular hypertrophy, sudden death, aortic insufficiency or endocarditis. Increasing left outflow tract gradient is a well-accepted indication for surgical treatment of discrete subaortic membrane, this indication may overlook the shelf’s progressive nature to grow and encroach on the leaflets of the aortic valve, potentially impairing future valve function. Since the morbidity and mortality for discrete subaortic membrane resection is negligible, resection may be indicated at the time of echocardiographic diagnosis to minimize aortic valve impairment [7, 8].

Transthoracic echocardiography is used to diagnose and follow of progression of all types of left outflow tract obstruction, especially discrete subaortic membrane; however, its ability to demonstrate the extent of discrete subaortic membrane encroachment onto the aortic valve is unclear.

The purpose of this study was to determine the sensitivity and specificity of transthoracic echocardiography in diagnosis of discrete subaortic membrane, to determine convenient time for surgical intervention, and for identifying involvement of the aortic valve by subaortic shelf.

## Material and Methods

A retrospective review of the medical records and echocardiograms of 18 patients [14 male (77%) and 4 female (23%)] with discrete subaortic membrane, aged 11 month to 12 years, with mean age of 5 years and 3 month, diagnosed at the Pediatric Clinic in Prishtina, during the period September, 1999 and December, 2010 were done. At the same time, database and medical reports for 13 patients (72%) undergoing primary repair for discrete subaortic stenosis in different European and North America centers, as a consequence that in Kosova still don’t exist cardiac surgery service; at the same time surgical reports of all operated children were analyzed. All operations were performed utilizing cardiopulmonary bypass. Access to the discrete subaortic membrane was achieved through a transverse aortotomy and, circumferential resection of the entire obstructing shelf was performed. 5 (27%) patients had isolated discrete subaortic membrane, 10 patients (55%) had CoA and 13 patients (72%) had bicuspid aortic valve, 2 (11%) patients had supravalvular aortic stenosis.

A three pediatric cardiologist executed minimum 3 echocardiograms in all children, with a mean interval of 6 month. None of the patients was receiving cardiovascular medications at the time of examination. All the echocardiograms were performed at rest, without sedation, and include M- and two dimensional modes, continual and color Doppler imaging. Used ultrasound systems were Acuson Aspen, Acuson Sequoia and Hewlett Packard Sonos 2000. The systolic function of the left ventricle was evaluated through the ejection fraction, using the M-mode measurements. The morphological aspect of the discrete aortic membrane, aortic valves and others heart structures was evaluated by the two-dimensional mode, while the severity of discrete membrane and aortic valves stenosis was determined by continual and color Doppler, according to the recommendations of the American Society of Echocardiography.

## Results

Four patients, in neonatal age were operated from critical coarctation of the aorta and, initial signs of congestive heart failure were presented. 2 of them were operated in Belgrade, Serbia and 2 in Losana, Zvicerland. In all of them bicuspid aortic valve was noted but, none of them subaortic membrane was registered. During the next 2 years of following all of them subaortic membrane manifested, while by continual and color Doppler increasing turbulent flow was registered.

The mean group of children in our study (12 patients 66%) subaortic membrane was diagnosed during the routine echocardiogarphic examination for the heart murmur. 6 of them had aortic coarctation and, 6 other had bicuspid aortic valve with consequence of aortic stenosis. In 2 oldest patients in our group, diagnosis was decided at tertiary level for cardiac evaluation, after routine examination from school doctor where, high blood pressure was noted. 2 patients had additional non cardiac congenital anomalies, with one patient each having Down and Alagille’s syndrome, a variant of Shone’s syndrome.

**Figure 1:**
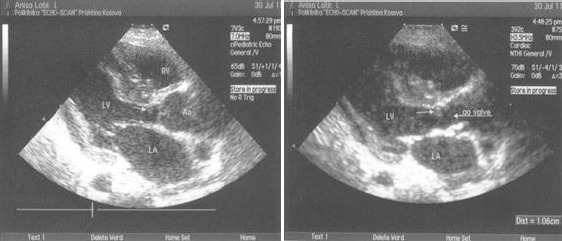
*Transthoracic long-axis view in diastole (Fig 1a), and in systole (Fig 1b) shows subaortic shelf, and as a consequence, hypertrophy of the left ventricle and dilated ascending aorta*.

In all patients pre-operative trans thoracic echocardiography demonstrated increasing velocity in left outflow tract with e mean peak of 3.9 m/s plus or minus 1.1 m/s with a mean peak left outflow tract gradient of 60.8 plus or minus 19.3 millimeters of mercury. Trivial aortic insufficiency was noted in 8 patients (44 %), mild in 6 patients (33%) and absent was in 4 patients (22%).

**Figure 2:**
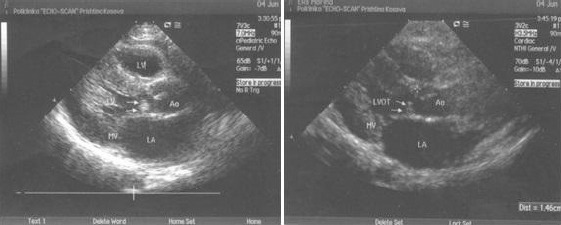
*Transthoracic view of eight years old boy, previously operated from perimembranous VSD, showed subaortic membrane few millimeters under arotic valve, closing left ventricular outflow tract*.

A total 13 patients undergoing primary repair of the discrete subaortic membrane, where intra-operative evaluation revealed involvement of the aortic valve by a discrete subaortic membrane in 10 patients or 77%.

**Figure 3:**
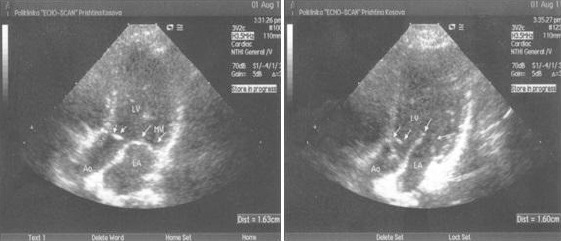
*Long-axis view in diastole (Fig 1a), and in systole (Fig 1b) shows hyperechogenic subaortic shelf, hypertrophic left ventricle and dilated left atrium as a consequence of severe mitral regurgitation*.

Reviewing our echocardiograms reports and reports from the centers where surgery was done, showed that the involvement of the aortic valve preoperatively was identified in 4 patients (40%). There was a significant difference between involvements of the aortic valve predicted by pre-operative echocardiogram reading compared with the involvement of the aortic valve documented intra operatively. The sensitivity and specificity of pre-operative trans thoracic echocardiography to diagnose involvement of the aortic valve are 40% and 77%.

## Discussion

A variety of lesion can obstruct the subaortic outflow tract and the lesion has been described in many ways and has been the subject of multiple investigations. Although often termed “membranous”, almost always the lesion is a firm fibrous shelf that encircles the outflow tract in diaphragmatic fashion. Discrete subaortic ridges are not congenital; they are acquired lesion as a result of some hemodynamic abnormality, and are not usually found before the age of 9 months. The membrane usually extends from the anterior septum to the anterior mitral leaflet, causing the variable degree of obstruction to flow. Lesion is associated with other sequels as aortic regurgitation, infective endocarditis, left ventricular hypertrophy and recurrence after surgical resection [9-12].

Symptoms are uncommon with this subvalvar stenosis, even when the narrowing is severe; however, occasionally syncope and giddiness occur. In undiagnosed and presenting in middle life, congestive heart failure, dyspnoea and syncope have been described. Clinical manifestation and physical signs are similar to aortic valvar stenosis (carotid thrill, left ventricular apex and aortic ejection systolic murmur).

Echocardiography with Doppler ultrasound is the quickest and best way of making the diagnosis and differentiating the common from rarer forms. M-mode echo allows identification of the degree of left ventricular hypertrophy, the presence of septal hypertrophy and characteristic mid-systolic vibration of aortic valve. Cross-sectional examination in the parasternal long axis shows the characteristic ridge in the outflow tract. With two-dimensional echocardiography, these membranes are seen as a discrete linear echo in the left ventricular outflow tract perpendicular to the intreventricular septum. Because the membranes are parallel to the beam, recording these structures from the parasternal long-axis window may require the use of multiple transducer positions. In many cases, the membranes are detected more easily from the apical views, where the ultrasound beam is oriented perpendicular to the structure [13-15]. In older children or young adults, transesophageal echocardiography demonstrates this lesion more fully and it’s a better way to identify associated lesions of the mitral and aortic valves. Two-dimensional echocardiography distinguished discrete subarotic membrane form subaortic fibromuscular ridge or tunnel. Tunnel-type subaortic obstruction, rarely seen in adults, is characterized by diffuse thickening and narrowing of the left ventricular outflow tract with associated concentric left ventricular hypertrophy [16-18].

Doppler imaging plays an essential role in the evaluation of these patients. After the location and orientation of the jet are visualized with color flow imaging, continuous wave can be used to estimate the peak pressure gradient across the membrane. In the absence of aortic valve stenosis, this value correlates well with the catheterization-derived measure of obstruction. In the presence of multiple serial stenoses, Doppler imaging may overestimate the catheterization-measured gradient [19, 20].

Because of progressive nature of the disorder, and the presence of aortic regurgitation, surgery is indicated even when the gradient through left ventricular outflow tract is over 30 – 40 millimeters of mercury. There are several controversies regarding the optimal surgical management of these patients, including additional indications for shelf resection, the timing of resection, and the indication for septal myectomy. Additional indications for resection at many centers include the presence of co-existing cardiac lesions and evidence of new or progressive aortic insufficiency. The progressive nature of discrete subaortic membrane is unpredictable, thus the timing of surgical intervention is controversial [21, 22]. Some centers recommend earlier surgical intervention to avoid the development of the left ventricular hypertrophy and aortic insufficiency. Scraping a fibrotic shelf of the aortic valve is technically challenging and puts the aortic valve leaflets at risks of perforation. At the same time, it is difficult to imagine that this damage to the endothelium of the aortic leaflets does not change the natural history of the valve’s function. There are several controversies regarding to the concomitant septal myectomy which aim is to achieve maximum relief of left ventricular outflow tract obstruction and perhaps reduce re-growth of the discrete subaortic membrane. Other centers reported little additional benefit with septal myectomy [23, 24].

**Table 1:** Baseline characteristics

Male	14 (77)
Age	11 m – 12 y
(Mean 5 y 3 m)	
Associated anomalies	13 (72)
Coarctation of the aorta	10 (55)
Bicuspid aortic valve	13 (72)
Supravalvular aortic stenosis	2 (11)
Surgical resection of subaortic membrane	13 (72)
Other surgeries performed during follow-up	
Coarctation repair	10 (55)
Supravalvular aortic repair	2 (11)
Konno procedure	3 (16)
Aortic valve replacement	2 (11)

This study demonstrates that the transthoracic echocardiography is the best noninvasive diagnostic procedure in diagnosis subvalvar aortic stenosis and estimation time for surgical intervention. At the same time echocardiography has either the sensitivity or specificity to demonstrate aortic valve encroachment by a discrete subaortic membrane. Some centers recommended transesophageal echocardiography as a routine method for following discrete subaortic shelf progression due to its invasive nature. From 13 our patients operated from subaortic membrane 10 of them had involvement of the aortic valve. 4 of them required re-operation after 3 - 6 years, where in 3 of them primary resection of membrane less than 2 years of age were done. All 4 reoperated patients had involvement of the aortic valve and after surgery moderate aortic insufficiency were manifested. In this series of 6 patients with moderate aortic insufficiency 2 patient developed late post-operative insufficiencies and aortic valve replacement were done. Since 4 patients in our study, who required re-resections, had the involvement of the aortic valve at the time of primary resection, the risk of next reoperation associated with the involvement of the aortic valve must be accounted for.

In conclusion, basing on the data of our study and recent published data from many centers we can conclude that:

- Transthoracic echocardiography is the best diagnostic procedure in diagnosis and assessment of the all forms of subaortic stenosis;
- Transthoracic echocardiography is not able exactly to demonstrate involvement of the aortic valve by discrete subaortic membrane and reliably to give prognosis of possible postoperative aortic insufficiency.
- Transesophageal echocardiography is more sensitive method in predication of the involvement of subaortic valve by discrete subaortic shelf but there are now still recommendations for improving this method as a routine modality.
- The increasing peak gradient of left ventricular outflow tract greater than 40 millimeters of mercury and age of patients above of 5 years are accepted as an indication for surgery. We can conclude also that the distance from discrete membrane to the aortic valve is an important predicting factor for involving the aortic valve by discrete membrane.
- Bicuspid aortic valve disease, coarctation of the aorta, and supravalvular aortic stenosis were associated with the need for aortic stenosis surgery. Careful clinical follow-up of this young population to monitor aortic valve status continues to be warranted even after a successful surgical resection.
